# Socioeconomic costs and health-related quality of life in juvenile idiopathic arthritis: a cost-of-illness study in the United Kingdom

**DOI:** 10.1186/s12891-016-1129-1

**Published:** 2016-08-02

**Authors:** Aris Angelis, Panos Kanavos, Julio López-Bastida, Renata Linertová, Pedro Serrano-Aguilar, BURQOL-RD Research Network, Pedro Serrano-Aguilar, Renata Linertová, Julio López-Bastida, Juan Oliva-Moreno, Manuel Posada de la Paz, Manuel Hens Pérez, Ignacio Abaitua, Domenica Taruscio, Yllka Kodra, Arrigo Schieppati, Rumen Stefanov, Georgi Iskrov, László Gulácsi, Márta Péntek, Rosa Sánchez de Vega García, Claudia Delgado, Panos Kanavos, Aris Angelis, Elena Nicod, Johann-Matthias Graf von der Schulenburg, Alexander Kuhlmann, Ulf Persson, Ola Ghatnekar, Karine Chevreul, Karen Brigham, Giovanni Fattore, Marianna Cavazza

**Affiliations:** 1 Medical Technology Research Group, LSE Health, London School of Economics and Political Science, London, UK; 2 University of Catilla-La Mancha, Talavera de la Reina, Toledo, Spain; 3 Red de Investigación en Servicios Sanitarios en Enfermedades Crónicas (REDISSEC), Madrid, Spain; 4 Canary Islands Foundation for Health and Research (FUNCANIS), Las Palmas de Gran Canaria, Spain; 5 Evaluation and Planning Service at Canary Islands Health Service, Santa Cruz de Tenerife, Spain

**Keywords:** Juvenile idiopathic arthritis, Rare diseases, Cost-of-illness, Socioeconomic cost, Health related quality of life, UK

## Abstract

**Background:**

Juvenile idiopathic arthritis (JIA) refers to a number of rare chronic inflammatory diseases. Although JIA imposes a significant societal burden, limited data are available on the cost of JIA. The study’s objective is to quantify the socioeconomic burden of JIA patients in the United Kingdom (UK), along with their health-related quality of life (HRQoL).

**Methods:**

A bottom-up, cross-sectional, cost-of-illness analysis of 23 patients was carried out. To collect data on demographic characteristics, health resource utilization, informal care, productivity losses and HRQoL, questionnaires were administered to and completed by patients or their caregivers. The EuroQol five dimensions (EQ-5D) instrument was used to measure HRQoL.

**Results:**

This study found that the average annual cost for a JIA patient was €31,546, with direct health care costs equalling €14,509 (46.0 % of total costs), direct non-health care costs amounting to €8,323 (26.4 %) and productivity losses being €8,715 (27.6 %). This was calculated using unit costs for 2012. The largest expenditures on average were accounted for by early retirement (27.0 %), followed by informal care (24.1 %), medications (21.1 %), outpatient and primary health care visits (13.2 %) and diagnostic tests (7.9 %). Important differences existed between JIA patients in need of caregiver assistance and those with no need (€39,469 vs. €25,452 respectively). Among adult JIA patients, mean EQ-5D index scores and visual analogue scale (VAS) scores were found to be 0.26 and 49.00 respectively; the same scores among caregivers were 0.66 and 67.14 respectively.

**Conclusion:**

JIA poses a significant cost burden on the UK society. Over half of the total average costs (54 %) are related to non-health care and productivity losses. HRQoL of JIA patients is considerably worse than the UK general population.

## Background

Juvenile idiopathic arthritis (JIA) refers to a group of different rare disorders, mainly manifesting as chronic inflammatory arthritis of unknown etiology. Onset of the disease occurs before adulthood, progressing to erosive arthritis and usually causing disability [1]. Subtypes of JIA include oligoarthritis (40 %), enthesitis-related arthritis (15 %), systemic arthritis (14 %), polyarthritis (14 %), psoriatic arthritis (1 %), and other arthritis types (16 %), with all subtypes having different phenotypes, courses and prognoses [2]. About one in every 1,000 children across the world suffers from JIA [3, 4]. Despite ethnic differences in prevalence [5] JIA is the type of chronic rheumatic illness children are most likely to suffer from, causing both short and long-term disability [6]. The international literature reports that children with JIA live with impaired functions due to joint swelling, pain and stiffness, with the possibility to experience an enduring disease activity and disability in adulthood and throughout their lifespans [7].

Available treatments for JIA patients aim to improve well-being while minimising side effects; first line treatments for the control of inflammation usually involve non-steroidal anti-inflammatory drugs and intra-articular glucocorticoid injections. Second line treatments usually include disease-modifying anti-rheumatic drugs (DMARDs), such as methotrexate (MTX) and tumor necrosis factor (TNF) alpha inhibitors. Predicting patients’ likely response to MTX can aid the prevention of side effects while saving time through prompt switch of patients to alternative therapies (e.g. biological drugs) in order to prevent irreversible complications [8, 9].

JIA imposes both a significant health burden on patients’, caregivers’ and families’ lives, as well as an economic burden on society due to the substantial health care costs associated with increased utilization [7, 10–14] while affecting health-related quality of life (HRQoL) [15]. Differences are reported among countries both in the overall magnitude and the main contributor of costs in JIA, whether across sub-types or for each sub-type. Agreement exists that total mean costs are highest for active seropositive polyarthritis and lowest for active enthesitis related arthritis [7, 10–14].

Data on the cost of JIA are less frequently available than for other illnesses, including lack of a comprehensive study in the United Kingdom (UK). In the present analysis we present primary JIA data at patient-level from the UK collected as part of the BURQOL-RD project [16]. Our objective is to provide critical insights into the JIA disease burden, first, by estimating the societal costs of the disease in terms of direct health care, direct non-health care and productivity losses, and, second, by assessing the HRQoL of patients and their caregivers based on the severity of their disease.

## Methods

### Research design and sample

The study involved non-institutionalised patients with a JIA diagnosis receiving outpatient care. A bottom-up, cross-sectional design was adopted. Patients were recruited from and contacted by the National Rheumatoid Arthritis Society (NRAS). Patient eligibility criteria included a JIA diagnosis, a non-institutionalised status and a membership with NRAS. Participants were asked to complete a survey, which was anonymous; none of the questions contained identification information.

### Information and variables of interest

A survey tool was administered to eligible patients by NRAS electronically and through post in February 2013. Two main sections comprised the questionnaire, one relating to costs and the other relating to HRQoL. Collection of data took place between February 2013 and April 2013. Data collected included demographic, clinical and resource use evidence.

Following data collection, patients were allocated into two groups for the analysis: a high-severity group that was in need of caregiver assistance to perform daily basic (e.g. dressing, eating, hygiene, etc.) or instrumental (e.g. laundry, meal preparation, shopping, etc.) activities and, a low-severity group, if no such assistance was needed.

### Costing methodology

A prevalence-based cost-of-illness approach was adopted in order to estimate the utilisation of resources and calculate costs from a societal perspective: direct health care resources, non-health care resources (formal or informal care), and productivity losses (i.e. indirect costs) were measured. Specifically, a bottom-up costing approach was adopted based on which average annual costs were estimated.

Resource use data were collected for all patients and, if appropriate, for caregivers as well. Our survey tool requested information for the period of 6 months preceding the start of the study (or 12 months for the case of hospital admissions), which were extrapolated to one year. For the calculation of productivity losses patients and caregivers provided information on working time reductions they experienced (sick leave or/and early retirement), with non-professional caregivers giving details on any informal care they provided.

Health care utilisation assisted in the calculation of direct medical costs by using unit costs and the respective mean patient utilisation to derive the cost of resources used. All information concerning resource utilisation data was obtained from the survey tool, including hospital admissions and emergency visits numbers together with related outpatient care volume (e.g. medical examinations and tests, health care professionals’ visits, home care and rehabilitation).

The UK payment by results database was used to collect data on unit costs [17], while other resources (publicly accessible) were used to complete gaps in the data [18, 19]. Annual costs per patient were derived by multiplying unit costs with the corresponding resource amounts for the year 2012. Likewise, resource consumption data concerning the utilisation of prescription drugs and medical devices were also collected through the surveys. For prescription drugs, in the case that units per pack information was unavailable, the largest dispensation package was assumed; the unit costs of drugs were collected from the National Drug Tariff database [20] and the British National Formulary [21], while medical devices unit costs were collected through online retails.

Direct non-health care costs were derived following the aggregation of the following cost types: non-health care transportation, social care services (i.e. formal care) and caregiver’s time (i.e. informal care^1^). As part of informal care, time spent helping patients with their basic activities of daily living (ADL) and necessary instrumental activities of daily living were also considered. The proxy good method was used to monetise the value of caring hours by applying a professional caregiving hourly rate to the informal caregiver’s time [22].

The social services category included evidence on formal (i.e. paid) care supplied by professional caregivers, along with other social services.

Productivity losses were derived by converting days of work absence (due to sick leave or early retirement) into economic cost by adopting a human capital approach [23], applying average national earnings data from 2012 [24].

### Patient & caregiver HRQoL

The EuroQol five dimensions (EQ-5D) instrument [25], the Barthel Index [26] and the Zarit Burden Interview [27] were used to collect patient and caregiver outcomes. The EQ-5D tool is a generic HRQoL instrument regularly used as part of health technology assessment (HTA). It consists of two components, the EQ-5D descriptive system which produces index scores from 0 to 1 (corresponding to the states of death and perfect health respectively) consisting of five dimensions (mobility, self-care, everyday activities, pain/discomfort and anxiety/depression), and the EQ-5D Visual Analogue Scale (VAS), comprised from a vertical 20-cm scale and ranging from 0 to 100 (denoting the worst and best imaginable health states respectively) [25]. For the UK general public, valuations for different health states have been modelled [28].

The Barthel Index is a measure of physical disability being extensively used to assess a person’s ability to complete ten basic ADLs, producing a numerical measure that reflects amount of dependence. For the UK, overall scores fluctuate between 0 and 20, with higher scores denoting lower disability, and therefore dependence, and vice versa [26].

The Zarit Burden Interview is a self-reported measure used to express burden among caregivers. Items on this instrument correspond to statements that caregivers respond to through a 5-point scale that ranges between 0 and 4, denoting *never* and *nearly always* respectively. Overall scores produced can fluctuate from 0 to 88, with >61 reflecting severe burden and scores <21 representing little or no burden to caregivers [27].

## Results

In total, 38 questionnaires were received from JIA patients out of the 62 questionnaires sent. Among these, 15 questionnaires were excluded because of insufficient or inadequate information, producing a valid sample of 23 patient questionnaires in total.

Patients’ characteristics are summarised in Table 1. There were ten adult patients and 13 adolescent patients with their average age being 21 years (average age of diagnosis was 5 years); 21.7 % of patients were males and 43.5 % of patients were supported by a caregiver with an average age of 43.1 years. The sample comprised the following disease sub-types: systemic-onset juvenile arthritis (Still’s disease) 30.4 % (*n* = 7), oligoarticular arthritis 26.1 % (*n* = 6), rheumatoid factor-positive polyarthritis 17.4 % (*n* = 4), rheumatoid factor-negative polyarthritis 8.7 % (*n* = 2), enthesitis-related arthritis (spondylarthropathies) 4.3 % (*n* = 1), juvenile psoriatic arthritis 4.3 % (*n* = 1), unclassified types 8.7 % (*n* = 2). The average total time spent on informal caregiving, denoting the existence of minimum one dedicated caregiver, was 20.8 h each week, (or 1,087 h per year).

**Table 1:** Sample characteristics of JIA patient sample (*n* = 23, SD in brackets)

Average age (years)	
All patients (*n* = 23)	21.4 (16.8)
Adult patients (*n* = 10)	38.5 (9.8)
Adolescent patients (*n* = 13)	8.3 (3.8)
Average diagnosis age (years)	
All patients (*n* = 23)	5.2 (4.4)
Adult patients (*n* = 10)	5.9 (5.2)
Adolescent patients (*n* = 13)	4.6 (3.9)
Sex	
Male	21.7 %
Female	78.3 %
Disease subtype	
Systemic-onset juvenile arthritis (Still’s disease)	30.4 %
Oligoarticular arthritis	26.1 %
Rheumatoid factor-positive polyarthritis	17.4 %
Rheumatoid factor-negative polyarthritis	8.7 %
Enthesitis-related arthritis (spondylarthropathies)	4.3 %
Juvenile psoriatic arthritis	4.3 %
Unclassified types	8.7 %
Is there a caregiver?	
Yes (*n* = 10)	43.5 %
No (*n* = 13)	56.5 %
Average age of (principal) caregiver (years)	43.1 (9.7)
Average informal care hours per week (whole sample)	11.8 (29.1)
Average informal care hours per week (if there is a caregiver)	20.3 (23.6)
Health Related Quality of Life (Visual Analog Scale)	
Adult JIA patients (*n* = 10)	49.00 (12.43)
*Visual Analog Scale score for general population* ^a^	*86.56 (13.79)*
Main Caregivers for JIA patients (*n* = 7)	67.14 (26.12)
*Visual Analog Scale score for general population* ^b^	*86.56 (13.79)*
Health Related Quality of Life (EQ-5D index score)	
Adult JIA patients (*n* = 10)	0.262 (0.239)
*EQ-5D index score for general population* ^a^	*0.91 (0.16)*
Main Caregivers for JIA patients (*n* = 7)	0.663 (0.367)
*EQ-5D index score for general population* ^*b*^	*0.91 (0.16)*
Average Barthel Index (*n* = 17)	16.2 (3.6)
Average Zarit scale (*n* = 8)	22.9 (6.6)

^a^Reflects general population social tariffs/utilities for the respective patients’ age group (i.e. 35–44)

^b^Reflects general population social tariffs/utilities for the respective caregivers’ age group (i.e. 35–44)

**Fig. 1:**
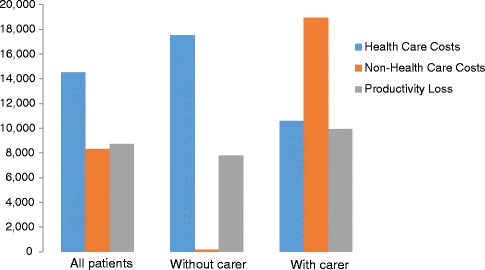
Average breakdown of costs across different patient study groups (2012, €)

**Fig. 2:**
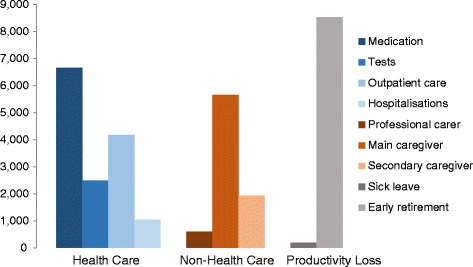
Average costs per JIA patient broken down by type of cost (2012, €)

In 2012, the average cost per patient was calculated at €31,546 (Table 2), about half of which was attributed to direct health care (HC) costs (46.0 %), and the other half being evenly divided between productivity losses (PL) (27.6 %) and direct non-health care (NH) costs (26.7 %) (Fig. 1). The largest HC cost category was prescription medications averaging €6,667 (46.0 % of HC costs, 21.1 % of overall costs) followed by outpatient and primary health care visits (28.7 % of HC costs, 13.2 % of overall costs) and diagnostic tests (17.2 % of HC costs, 7.9 % of overall costs) (Fig. 2). Informal care constituted the highest NH costs, with €7,601 on average (91.3 % of NH costs, 24.1 % of overall costs), largely relating to main caregiver costs (68.0 % of NH costs, 17.9 % of overall costs). Professional carer costs comprised 7.2 % of NH costs and 1.9 % of overall costs, whereas non-health care transport amounted to 0.9 % of NH costs and 0.2 % of overall costs. Social services represented 0.6 % of NH costs and 0.2 % of overall costs. Finally, early retirement amounted to €8,526 accounting for 97.8 % of PL and 27.0 % of overall costs, whereas sick leave accounted for just 2.2 % of PL and 0.6 % of overall costs.

**Table 2:** Average annual costs per JIA patient (2012, in €)

	Total (*n* = 23)		Without Carer (*n* = 13)		With Carer (*n* = 10)	
	Mean	SD	Mean	SD	Mean	SD
Health Care Costs						
Medication	6,667	9,206	9,105	11,060	3,496	4,931
Tests	2,493	2,662	3,114	3,335	1,686	1,101
Outpatient and primary health care visits	4,169	3,206	3,718	3,355	4,754	3,074
Acute hospitalisation	1,047	2,962	1,451	3,894	522	826
Devices	114	162	100	131	132	201
Health care transportation	19	92	33	120	0	0
Subtotal	14,508	14,877	17,523	18,331	10,590	7,926
Non-Health Care Costs						
Social services	50	241	89	320	0	0
Professional carer	597	2,546	0	0	1,372	3,830
Non-health care transportation	75	143	59	146	96	145
Caregiver time costs (informal care)	7,601	18,703	0	0	17,481	25,751
Main caregivers	5,661	11,722	0	0	13,020	15,147
Secondary caregivers	1,940	9,027	0	0	4,462	13,663
Subtotal	8,323	18,665	148	338	18,950	25,089
Total Direct Costs (Health Care Costs & Non-Health Care Costs)	22,831	24,728	17,671	18,550	29,540	30,862
Productivity Loss						
Sick leave	190	513	240	600	125	395
Early retirement	8,525	14,673	7,542	14,332	9,804	15,786
Subtotal	8,715	14,566	7,782	14,636	9,929	15,705
TOTAL COSTS	31,546	28,568	25,452	27,146	39,469	29,817

Whether or not a JIA patient needed caregiver assistance, impacted greatly the results. A JIA patient with caregiver assistance accumulated an average overall cost of €39,469 per year, compared with €25,452 for a patient where caregiver assistance was not needed. In the presence of a caregiver, informal care was the greatest cost attribute, averaging €17,481 (44.3 % of overall costs). Direct HC and NH costs respectively represented 26.8 % and 48.0 % of overall costs. With regards to HC cost types, outpatient and primary health care ranked at top (44.9 % of HC costs, 12.0 % of overall costs), followed by medications (33.0 % of HC costs, 8.9 % of overall costs), and diagnostic tests (15.9 % of HC costs, 4.3 % of overall costs). Professional care amounted to 7.2 % of NH costs and 3.5 % of overall costs. A loss in productivity amounted to 25.2 % of overall costs, with early retirement accounting for 98.7 % of PL and 24.8 % of overall costs respectively.

Where a carer was not required, HC and NH costs amounted to 68.9 % and 0.6 % of overall costs respectively, with PL amounting to 30.6 % of overall costs. The greatest attributes of HC costs were medications (52 % of HC costs, 35.8 % of overall costs), outpatient visits (21.2 % of HC costs, 14.6 % of overall costs) and diagnostic tests (17.8 % of HC costs and 12.3 % of overall costs). Only 0.4 % and 0.2 % of overall costs were attributed to social health services and non-health care transport respectively. Finally, sick leave and early retirement attributed 3.1 % and 96.9 % of PL and 0.9 % and 29.6 % of overall costs respectively.

In terms of adult patients’ HRQoL, the mean EQ-5D index score was estimated to be 0.26 and the mean EQ-5D VAS score was 49 (out of 1.00 and 100 respectively) (Table 1). Both scores were below the respective age-adjusted values of the UK public (0.91 and 86.56 respectively) [29]. Similarly, the mean EQ-5D index and VAS scores for carers were 0.66 and 67.14 respectively (Table 1), both being considerably lower than the age-adjusted UK public values. The average Barthel index among adult patients was 16.2 associated with moderate dependence, whereas the average Zarit interview burden score was 22.9, suggesting a mild to moderate burden for those in care of patients (Table 1).

## Discussion

The above findings suggest that JIA is likely having a considerable economic impact on the UK, especially with regards to non-health care costs representing more than half of overall average costs, and with the HRQoL of JIA patients being substantially below public valuations.

Among the low prevalence diseases and orphan indications space, JIA represents a clinical condition with important societal impact and significant cost burden in high-income countries, including in the UK. Its prevalence, incidence, mortality and morbidity combined with a high socioeconomic impact, i.e. cost and HRQoL, requires attention from health authorities.

There is a lack of socioeconomic research into the burden of JIA. A recent systematic literature review indicated nine available studies on JIA costs [30]. JIA’s economic impact was investigated in seven of the studies [2, 13, 14, 31–34], one study looked into etanercept’s (TNF-alpha inhibitor) impact on overall costs [35], and another investigated JIA hydrotherapy’s cost-effectiveness [36]. Some of the studies also highlighted the significant costs associated with JIA while at the same time showing substantial variation between subgroups. Measured in 2012 €, average overall cost per year was calculated at €4,143 (€3,471 in 1999) [2] and €29,613 (US $33,171 in 2000) [35] per patient, indicating that 80 % of total costs may be caused by only a fraction of patients, and over 50 % of direct costs may stem from the treatment of only a small proportion of inpatient cases [2]. Loss of parents’ income has been used by Minden and colleagues to reflect indirect costs in Germany, initially estimated annually for each patient at €1,870 or 86 % of direct costs in 1999, subsequently declining respectively to €274 or 6 % of direct costs annually in 2008 [34], possibly due to the introduction of more effective drug therapies (TNFα inhibitors) enabling caregiving parents to return to work. Indeed, a Finnish study showed a reduction of 50 % in indirect costs at €1,507 following the introduction of TNFα inhibitors, associated with an additional €3,767 direct costs per patient annually [35], and another study in the UK setting estimated indirect costs much lower at €142 per patient per year (5 % of direct costs) [36]. In respect to direct costs, these have been estimated at €4,464 (€4,403 in 2008) [34] with other estimates for direct healthcare costs ranging from €2,202 (GBP 1,649 in 2005) [13] to €9,273 (US $7,904 in 1992) [31], and the greatest attribute being etanercept treatment at €27,603 (US $30,919 in 2000) accounting for up to 54 % of direct costs [35]. Depending on the study, direct costs accounted between 6 % and 55 % of total costs, with health care costs comprising 95 % of direct costs, and 60 % of health care costs accredited to outpatient visits with the remaining 40 % to inpatient [2, 34]. Low expenditure on medical devices compared to early 2000s might be partly attributed to changing patterns of care, as reflected through the shift from application of artificial joints (AJs) in adult patients, which is now a rare event, to treatment with DMARDs, including MTX and TNF-alpha inhibitors. Overall, costs were shown to be skewed towards active patients with mean total costs being over seven times higher for patients in remission [2], and with a substantial sub-group variation.

Of all studies, only two addressed the UK context [13, 36] and only one measured productivity losses by using parents’ time away from work [36].

This study represents the first UK attempt to quantify the total cost for JIA patients while measuring direct non-healthcare costs. The present research draws attention to the significance of examining the monetary impact of JIA from a societal standpoint and the need for a cross-country understanding. The study’s results provide us with knowledge on how JIA costs are distributed and also the strain such costs add to the healthcare system, together with the impact on patient and caregivers expenses. The overall average cost of €31,546 per year (€25,452 to €39,468 respectively for patients without and with caregivers), is associated with significant expenditures related to early retirement (27.0 % of overall cost), informal care (24.1 %), medications (21.1 %), outpatient and primary health care visits (13.2 %) and diagnostic tests (7.9 %).

The relatively greater impact of informal care and indirect costs in our results compared with earlier studies may have several explanations, which, ultimately, can be attributed to the methodology adopted. For example, although earlier cost-of-illness studies have generally incorporated employment loss and informal care as part of indirect costs, later studies adopt overwhelmingly more detailed societal cost categories. In our study we have observed high (informal) care and high early retirement costs, the latter possibly due to the relatively young age of the patient population (38.5 years). Other studies on JIA have proxied indirect costs through productivity losses of the caregiver [34, 36]. Our results are likely underestimating real JIA costs due to the exclusion of institutionalised patients and any long-term care costs. Finally, by quantifying costs attributed to informal caregiving, our study shows informal care to be a major cost to UK society, which could be regarded as ‘hidden’ cost given the absence of acknowledgement in other studies.

For priority-setting and evaluation of health care interventions, HRQoL has been found to be a valuable measure in combination with other epidemiological, clinical and economic evidence on incidence, prevalence, mortality and costs. Our results illustrate that mean EQ-5D index scores both for adult JIA patients (0.26) and caregivers (0.66) are substantially lower than the (age-adjusted) equivalent in the general population (0.91), indicating lower quality of life. Despite its low prevalence, JIA results in a considerable monetary burden, with higher dependency patients being associated with greater productivity losses than lower dependency patients.

Our study has a number of drawbacks, as for example issues relating to sampling. The adopted recruitment process and study sample may reduce results’ external validity; although there were almost even proportions of high- and low-severity patients in our sample, selection bias is frequent among studies of rare diseases where few patient numbers is the general norm. Second, given that patient reported information was collected via a survey tool there might be potential recall bias and a 6-month recall period would not exclude this possibility. A third limit results from the exclusion of any JIA-specific HRQoL tools, such as the Juvenile Arthritis Quality of Life Questionnaire [37–39] or Arthritis Impact Measurement Scales [40]. Nevertheless, recently a systematic literature review on the use of various HRQoL instruments in the space of rare diseases suggested that the EQ-5D could be regarded as a valid measure for generic health outcomes in JIA disease progression [41]. Lastly, we employed cross-sectional data; ideally we should have adopted a prospective longitudinal study design, however this was not possible and to our best knowledge no such analysis has yet been carried out to study JIA.

Regardless of its limits, we believe our work is the most comprehensive and representative costing exercise on the burden of JIA in the UK to date, with the main advantage centring on the adoption of a bottom-up costing approach over a one-year period.

## Conclusion

This bottom-up aggregate cost and HRQoL study suggests that in the UK there are substantial health care costs due to JIA, nonetheless other societal costs, such as early retirement and informal caregiving, are proportionately greater, with disability being associated with significantly higher JIA societal costs. Overall, JIA presents an important societal cost being linked to a substantial HRQoL deterioration. These findings could be considered when examining treatment options for JIA sufferers and support program options for patients and caregivers alike. The study results could support the formation of integrated approaches for the periodic evaluation of rare diseases’ novel treatments and public policies impact, both within the UK and at a broader supranational level.

## Abbreviations

ADL, activities of daily living; DMARDs: disease-modifying anti-rheumatic drugs; EQ-5D, EuroQol Five Dimensions; HC, health care; HRQOL, health-related quality of life; JIA, juvenile idiopathic arthritis; MTX, methotrexate; NH, non-health care; NRAS, National Rheumatoid Arthritis Society; PL, productivity losses; TNF, tumor necrosis factor; UK, United Kingdom; VAS, visual analogue scale

## Appendix: BURQOL-RD research network

- Canary Islands Foundation for Research and Health (FUNCIS) (Spain): Pedro Serrano-Aguilar, Renata Linertová
- Universidad Castilla-La Mancha (Spain): Julio López-Bastida, Juan Oliva-Moreno
- Research Institute for Rare Diseases, Instituto de Salud Carlos III (Spain): Manuel Posada de la Paz, Manuel Hens Pérez, Ignacio Abaitua
- National Center for Rare Diseases, Istituto Superiore di Sanità (Italy): Domenica Taruscio, Yllka Kodra
- Mario Negri Institute for Pharmacological Research (Italy): Arrigo Schieppati
- Bulgarian Association for Promotion of Education and Science (Bulgaria): Rumen Stefanov, Georgi Iskrov
- Centre for Public Affairs Studies Foundation (Hungary): László Gulácsi, Márta Péntek
- Federación Española de Enfermedades Raras (Spain): Rosa Sánchez de Vega García, Claudia Delgado
- London School of Economics and Political Science (UK): Panos Kanavos, Aris Angelis, Elena Nicod
- Leibniz University Hannover (Germany): Johann-Matthias Graf von der Schulenburg, Alexander Kuhlmann
- The Swedish Institute for Health Economics (Sweden): Ulf Persson, Ola Ghatnekar
- University Paris Est (France): Karine Chevreul, Karen Brigham
- Universita Commerciale “Luigi Bocconi” (Italy): Giovanni Fattore, Marianna Cavazza

## Footnotes

1 Such care is provided by non-professional caregivers who are not remunerated, primarily being relatives, friends or neighbours of patients.
